# Anaemia and nutritional status of adolescent girls in Babile District, Eastern Ethiopia

**DOI:** 10.11604/pamj.2016.24.62.6949

**Published:** 2016-05-13

**Authors:** Kedir Teji, Yadeta Dessie, Tesfaye Assebe, Meyrema Abdo

**Affiliations:** 1 Haramaya Univesity, College of Health and Medical Science, East Harerge, Ethiopia; 2 Rift Valley University, Faculty of health sciences, Adama branch, Jimma, Ethiopia

**Keywords:** Adolescent, anemia, BMI for age, stunting

## Abstract

**Introduction:**

Nutritional status during adolescence plays an important role in the human lifecycle that influences growth and development and during this period nutrient needs are the greatest. The objective of this study is to assess anaemia and nutritional status of adolescent girls in the Babile district, Eastern Ethiopia.

**Methods:**

Data were collected from 547 adolescent aged 10-19 years by cross sectional study design. WHO Anthro-plus software was used to analyse Nutritional statuses of adolescents and magnitudes were determined using WHO 2007 references point. Haemoglobin was measured on site by hem cue machine. Descriptive and inferential statistical analysis was carried out depending on the nature of variables.

**Results:**

The result of the study show that 21.6% thin, 4.8% were over weighted and 1.1% was obese, 32% were anaemic and 15% of adolescents were stunted/ short stature than normal. Nutritional status of adolescent were low both in urban and rural adolescents, but severe thinness were higher among of rural (39.3%) compared to urban (37.5%) adolescents. Factors independently associated with stunting were place of residence, father occupation source of drinking water and age of the adolescents.

**Conclusion:**

Nutritional status of adolescent girls contributes to the nutritional status of the community. There is a need to initiate intervention measures to improve the nutritional status of adolescent girls who are the future ‘mothers-to-be’. Hence, there is a need to create awareness among adolescents and their family about nutrition and health.

## Introduction

World Health Organization (1986) defines adolescents as persons aged 10-19 years. This age group make up roughly 20% of the total world population and this period is transition from dependent childhood to independent adulthood. Physical growth at adolescent occurs earlier and is more rapid than during pre-adolescence [1]. Twenty percent of the final adult height and 50% of adult weight occurs during this period: bone mass increase by 45%, dramatic bone remodelling and soft tissues, organs, red blood mass increase in size [2].

The world health organizations, declared that, adolescent remain largely neglected, difficult to measure, hard to reach population in which the need of adolescent girls in particular often ignored [3]. Nutritional status during adolescence plays an important role in the human life cycle [4]. The diet of children and adolescent must be adequate to support normal and sometimes very rapid growth and development [5]. Nutrition in general influences the growth and development throughout infancy, childhood and adolescence; it is, however, during the period of adolescence that nutrient needs are the greatest [6]. Remarkably, 84% of the adolescents are in developing countries [3, 7]. Adolescent constitutes about 48% of Ethiopian population and about 25 percent of this age group is girls [8].

Among adolescents, girls constitute a vulnerable group, particularly in developing countries where they are traditionally married at an early age and exposed to a greater risk of reproductive morbidity and mortality [9]. The health and nutritional status of the adolescents’ girls are the reflection of cumulative effect of physical growth, the onset of the menarche and an increase in fat and muscle mass which place extra nutrition requirement. Physical growth of the adolescent girls’ generally related to their dietary intake which is determined by the availability of food in terms of quantity, quality and the ability to digest and absorb and utilize the food. Furthermore, under-fed girls are at risk of being stunted mothers who are likely to suffer obstetric complications and to deliver low birth weight babies[10]. In the absence of effective nutritional interventions, the low birth weight girls become the next generation of stunted mothers, thus, perpetuating the vicious cycle of malnutrition. Finding from Guatemala study on maternal-child-pair revealed that maternal birth size was a significant predictor of child's birth size and Child's birth weight increased by 29 g/100 g increase in maternal birth weight and child's birth length increased by 0.2 cm for every 1 cm increase in mother's birth length [11]. Studies from Asia [12–17] and few studies from African countries [18] have reported variances in prevalence of under nutrition that all of them signify adolescent malnutrition is a prevalent problem among the study population. Few studies in Ethiopia found that, under nutrition was common problem among adolescent girls, [19–21]. So, the current study aims at assessing magnitude of anaemia and nutritional status of adolescent girls in Babile district in Eastern Ethiopia.

### Methods

The study was conducted in Babile district which 560 km away from capital city Addis Ababa and 35 km away from Harar on eastern part of Ethiopia. The district has 21 kebele (smallest administrative unit in Ethiopia) with different climatic zones and rural urban mix living residences. Data were collected from low land agro ecological zones where the altitude is 1200 meter below sea level. Among 21 kebele, two of them are urban and 19 of them are rural. A total of about 98,341 populations found in the district according to 2007 CSA. Feeding style of the community were published elsewhere [22]. The present study was conducted during the period Dec 2012- February 2013 during food surplus season of the years.

Community based cross sectional study design were employed among adolescents’ girls aged 10-19 years old residing Babile district are the source population. The Study population were selected randomly in households who had adolescent girls aged 10-19 years old in the selected kebele of the district that were included in to the study randomly. The sample size for the study was calculated by taking level of significance to be 95%, (Z&/2=1.96), margin of error 3%. Since magnitude of Malnutrition among adolescent's girls, 50% prevalence was taken because no studies were found in the similar area. The final samples were 600 adolescents.

#### Sampling technique

Out of 21 kebele, the study was conducted among one urban and ten kebele from rural kebele were included in the study. By considering the list of the households as sampling frame and taking the adolescent in the households as sampling unit, simple random sampling was employed in order to select the households. Proportionality of the number of adolescent in each kebeles was also assumed. If adolescent girl/s is/are not found in the house, proximal household was included. In cases where there are two or more adolescents in the same households, one of them was selected randomly by lottery method. Adolescents in the age 10-19 years with severe illness and who are unable to speak were excluded from the study.

For the data collection; questionnaire, anthropometric and Haemoglobin assessment were used. Questionnaire consisting of different components (socio demographic/economic characteristics, food intake pattern and, food security status (both house hold and individual), dietary intake characteristics, morbidity status, physical activity, psychological related questions and substance use behaviour (khat chewing, smoking and others)) that are extracted from different literatures were used as data collection tool. The questionnaire was prepared in English and translated to local languages ( Afan Oromo) and then translated back to English to check the consistency.

A questionnaire developed by FANTA were used to measure Dietary diversity [23, 24]. A simple questionnaire designed to allow all types of foods consumed during each of the 24 previous hours to be noted. Anthropometric assessment constitute: Weight, Height, MUAC measurements. Weights were measured using digital weighting scale and recorded to the nearest 0.1 kg. Heights were measured using a locally produced wooden measuring board and recorded to the nearest 0.1 cm. Subjects were measured with minimal (light) clothing and removed their shoes or hats during the measurement [25]. Mid Upper Arm Circumference (MUAC) was measured by using insertion type MUAC tape used commonly for measurement of pregnant mothers. Stunting or short stature among adolescents (10-19 years of age) is defined as height-for-age less than -2 Z-scores of a reference

A portable battery operated photometer (HemoCue) was used to measure the haemoglobin values. Haemoglobin was measured by drawing drops of blood from the finger prink. A HemoCue-Cuvatte was filled with drop of blood and result read with in the 10 minutes of time. The photometer was calibrated before every session using provided standard. The method was recommended for epidemiologic studies in resource poor setting [26]. Both interview and blood sample collection was take place respectively at time of data collection from each client in a separate room. Haemoglobin level determination was done by trained laboratory technicians that are working outside of the respective district. And the measurements were adjusted for altitudes [27]. The cut-off point for anaemia was based on WHO (2011) recommendation for mothers and categorized as mild anaemia (Hgb 10.0-11.9 g/dl), moderate anaemia (Hgb 7.0-9.9 g/dl) and severe anaemia (Hgb less than 7.0 g/dl) [27].

Data were collected by laboratory and nurse diploma professionals after training was given. The data collectors were trained intensively on the data collection procedures, the context of specific questions across the questionnaire, anthropometric measurement procedures. For Haemoglobin determination two laboratory technicians to were employed to collect and process the sample of blood.

To control quality of data questionnaire was prepared first in English then translated Afan Oromo language then back to English to maintain its consistency. Four day training was given for the data collectors and supervisor about the objectives, methodology and process of the data collection by the principal investigator. The questionnaires were pre-tested among 5% of the total sample size in Haramaya district (out of study site). Based on the pre-test, validity and reliability of the measurement was checked, questions that pose difficulty were revised, edited, and those found to be unclear or confusing was removed. Each data collector obtained an opportunity to be acquainted with the interview and measuring technique. Two different measurements were taken for the height, weight and MUAC by two different measurement takers for every study subjects so that the average of the two were considered for the analysis. This would help in reducing the occurrence of measurement errors by single individual measurement. The principal investigator and supervisors were compiling the completed questionnaire every day and check them for inconsistencies, incompleteness and omissions. Any filled questionnaire which has a defect was rejected from the study. Moreover, data were double entered to check for data entry errors and correctness. The principal investigator was responsible for co-ordination and supervision of the overall data collection process.

The data were entered to Data EPI data and cleaning and editing was under taken before analysis. For the analysis SPSS (v 16.0) statistical packages was used for the analysis of the study. Descriptive statistics: Frequency, mean, standard deviation, and correlation was computed for the interest variables. Normality was checked by different plots (P-P and/or Q-Q-plot) if normality will not be maintained (food, meal frequency, anthropometric and biochemical analysis), in place of mean median was considered. Anthropometric data were entered and analysed using WHO Anthro-plus software. Bivariate and multivariate binary logistic regression regressions were applied when the variables are normally distributed. P-values of 0.05 were used as cutting points to determine significance of the variables

Ethical clearance was obtained from Haramaya University Institutional Research Ethics Review Committee. The data collectors were explained the objectives and benefits of the study to get informed oral consent from the study subject's family prior to data collection. When the study subject less than 18 years, we got informed oral consent from the study subject's family prior to data collection and additional verbal incent is obtained from the study subject when the study subject is less than 18. The respondents was told as they have the right to refuse or decline from the study at any time and refusing to participate on the study couldn't bring any effect on them.

## Results

In this study, out of 600 samples selected, a total of 547(91%) adolescents girls were include in the analyses. Main reasons for refusal were fear of injection to measure haemoglobin. The study population consisted of adolescent girls aged 10-19 years at the time of the survey. Of which 34.2% are from age 10-14 years old. The mean age of the subjects was 14.95(Std. Deviation of 1.04, majority 409(74.8%) of the participants were had little/ no household hunger, nearly half (50.6%) of them are from urban residence and only 23% of the community uses pipe water. The socio-demographic characteristics of the study population with level of anaemia distribution are depicted in the following table (Table 1).

**Table 1:** Descriptions of the respondent's socio-demographic condition of adolescent girl in Babile district of eastern Ethiopia, 2013

*Variables/ Characteristics N=547*	*Responses*	*N*	*%*	Anaemic	Non anaemic	X2	P –values
**Age**	10-14	187	34.2	43	144	0.6	0.001^+^
	15-19	360	65.8	132	228		
**Household hunger scale**	Little/no hunger	394	72.0	120	289	5.2	0.02^+^
	Moderate/severe hunger	153	28.0	55	83		
**Stunted**	Yes	82	15.0	27	55	0.39	0.84
	No	465	85.0	148	317		
Skipped meal	Yes	260	47.5	92	168	2.6	0.1
	no	287	52.5	83	204		
**Place of residence**	Urban	277	50.6	74	203	7.2	0.007^+^
	Rural	270	49.4	101	169		
**Their family status**	Both parent alive	450	82.3	151	299	2.8	0.91
	Loss of their family	97	17.7	24	73		
**occupation of respondents father**	Farmer	185	33.8	65	120	1.2	0.26
	Others	362	66.2	110	252		
**occupation of respondents mothers**	Farmer	147	26.9	60	87	7.2	0.007^+^
	Others	400	73.1	115	285		
**Family monthly expense greatly depend on**	Father	119	23.8	37	82	0.05 0.14 1.00	0.83 0.7 1.00
	Mother	93	18.6	28	65		
	Both of them	289	57.7	93	196		
**Source of drinking water**	Pip water	126	23.0	40	86	0.01	0.95
	Others	421	77.0	135	286		
**BMI for age z score**	Thin	118	21.6	38	80	0.02	0.88
	Normal	397	72.6	125	272	1.00	1.00
	Overweighed/obese	32	5.9	12	20	0.49	0.48

### Prevalence of anaemia and under nutrition among adolescent girls

Among 547 adolescent involved in the study 32% were anaemic. Out of which 1.8% had severe anaemia (haemoglobin level less than 7gm/dl). Distribution of nutritional status of respondent's was determined using the Using the 2007 WHO growth reference, classifications of BMI for age were as follows; 6.6% were very thin (< - 3SD), 15.0% were thin (≥ - 3SD and < -2SD), 72.6% have normal range (>-2SD and less than +SD), 4.8% were overweight (+ 1 SD and + 2 SD), and 2(1.1%)of them were obese (> + 2SD). Similarly among study adolescent 15.0% were stunted/have shorted stature (Table 2).

**Table 2:** Prevalence of anaemia and under nutrition among adolescent girls in Babile district of eastern Ethiopia, 2013

Reference values	N	%	95% CI
**MUAC**			
<18.5(severely malnourished)	107	19.6	16.3-23.1
18.5-22.5(moderately malnourished)	309	56.5	53.2-60.7
>22.5(normal)	131	23.9	20.4-27.8
**Overall Undernourished (<22.5)**	**416**	**76.1**	72.2-79.6
**BMI for age z score**			
<-3 z score(Sever thinness)	36	6.6	4.6-8.9
-3 to -2 (Mild thinness)	82	15.0	12.3-18.6
>-2 to<0.99 (Normal)	397	72.6	68.6-76.3
1.00 to 1.99 (Overweight)	26	4.8	3.1-6.9
>=2 (Obese)	6	1.1	0.4-2.3
**Haemoglobin**			
<7 (Sever Anaemia)	10	1.8	0.8-3.3
7-9.9(moderate anaemia)	21	3.8	2.4-5.8
10-11.9(mild anaemia	144	26.3	22.7-30.3
>12(normal range)	397	72.6	68.6-76.3
**Overall anaemia (HGB<12)**	**175**	**32.0**	24.8-32.5
**Stunting level**			
Stunted	82	15.0	12.1-18.3
Not stunted	465	85.0	81.7-87.9
**Height**			
120-150 cm	107	19.6	16.3-23.1
>150 cm	440	80.4	76.9-83.7

Adolescent girls between the age 17 and 19 years were more thin (29.5%), followed by age 16 years. Similarly stunting was higher among age 17-19 ages group and lowest were recorded among age 15. In the present study, level of anaemia were higher among age 16 years and followed by age 17-19 (Table 3).

**Table 3:** Nutritional status of the girls by age and residence of adolescent girl in Babile district of eastern Ethiopia, 2013

Age		Age of the adolescents			
		10-14	15	16	17-19
Nutritional status	Thinness(<-2SD)	46(24.6)	44(17.0)	15(26.3)	13(29.5)
	Normal range (>-2 to<0.99)	125(66.8)	204(78.8)	38(66.7)	30(68.2)
	Overweight and obese (≥+1SD)	16(8.6)	11(4.2)	4(7.0)	1(2.3)
Level of stunting	Stunted	37(19.8)	12(4.6)	18(31.6)	15(34.1)
	Non stunted	150(80.2)	247(95.4)	39(68.4)	29(65.9)
Level of anaemia	Anaemic	43(23.0)	85(32.8)	31(54.4)	16(36.4)
	Non anaemic	144(77.0)	174(67.2)	26(45.6)	28(63.6)

### Factors associated with nutritional status

In a bivariate logistic regression model variables with significant associations were identified. Finally those variables which have association in bivariate models were taken to multivariate logistic regression to compare the independent associations for solving cofounding effects of the variables. In multivariate logistic regression; place of residence, father occupation, thin, age of adolescent and source of drinking water were significantly associated with stunting. This study showed adolescent living urban were 57% less likely stunted compared to adolescent livening in rural (AOR=0.428, p=0.006) adolescent from farmer father were more 2.4 times more likely stunted compared to adolescents whose fathers were not farmer (AOR= 2.4 95%CI= 1.2-4.8) adolescent aged 10-14 were 48% less likely prone to stunting than aged 15-19(AOR= 0.52 95%CI= 0.30-0.90). Similarly this study revealed that those thin adolescents are more likely stunted compared to their counterparts (AOR= 3.5 95%CI= 2.1-6.1) (Table 4).

**Table 4:** Factors associated with adolescent stunting in Babile district, eastern Ethiopia

		Nutritional status						
Variable	Response	Stunted	Normal	COR	95%CI	AOR	95%CI	p-value
Place of residence	Urban	23	254	0.32	0.19-0.54+	0.428	0.23-0.78	0.006++
	Rural	59	211	1.00	1.00	1.00	1.00	
Family alive	Both parent alive	69	381	1.17	0.62-2.22	-	-	-
	One/both died	13	84	1.00	1.00			
Source of water	Pip water	17	92	1.06	0.59-1.89	1.9	1.0-3.5	0.05++
	Others source	65	373	1.00	1.00	1.00	1.00	
Father education	Illiterate	32	107	2.14	1.31-3.51+	1.08	0.57-2.1	0.7
	Read and write	50	358	1.00	1.00	1.00	1.00	
Mother education	Illiterate	50	211	1.88	1.16-3.04+	1.2	0.66-2.2	0.54
	Read and write	32	254	1.00	1.00	1.00	1.00	
Father occupation	Farmer	49	136	3.59	2.21-5.83+	2.4	1.2-4.8	0.009++
	Others	33	329	1.00	1.00	1.00	1.00	
mother occupation	Farmer	36	104	2.72	1.67-4.42+	1.2	0.6-2.4	0.57
	Others	46	361	1.00	1.00	1.00	1.00	
Wasting status	Thin/wasted	37	81	3.9	2.37-6.41+	3.5	2.1-6.1	0.000++
	Not wasted	45	384	1.00	1.00	1.00	1.00	
Anaemia level	Anaemic	27	148	1.05	0.64-1.73	-	-	-
	Non-anaemic	55	317	1.00	1.00			
Age	10-14	43	132	0.52	0.34-0.77+	0.52	0.30-0.90	0.018++
	15-19	144	228	1.00	1.00	1.00	1.00	
Household hunger scale	Little/no hunger	56	338	0.81	0.49-1.35	-	-	-
	Moderate/sever hunger	26	127	1.00	1.00			
Skipped meal yesterday	Yes	47	213	1.6	0.99-2.55	-	-	--
	No	35	252	1.00	1.00			

+ Significant by bivariate analysis

++ Significant by multivariable analysis

## Discussion

A strength of this study was it is community based study and the random selection of the households. Generalization may be made to the study communities as an attempt was made to identify randomized households and adolescents from the study communities. The major limitation of this study was the failure to collect information related to pubertal landmarks. Another limitation of the study was that the cross-sectional design makes any inference of growth pattern over time difficult. The cross-sectional nature of the study could only generate a hypothesis about the possible role of certain independent variables on the nutritional status of these adolescent girls but not their causal relationships.

This study have been documented that anaemia and nutritional states of Adolescents is an important concern for public health in Ethiopia. Adolescent is a transitional period between childhood and adulthood, targeting adolescence can provide an opportunity to prevent the onset of nutrition related chronic diseases in adults life, addressing adolescence specific nutrition issues and possibly also correcting some nutritional problems originating in the past.

This study revealed that nearly one third (32%), of adolescents were anaemic. Of which 1.8% had severe anaemia. The magnitude of anaemia among this adolescent was less than prevalence reported India (61%) [28] and almost similar to lactating mothers in the study area during food surplus season (34.2% [29] and by far higher than study conducted in Indonesia (9.7%) [30].

Prevalence rate of thinness among this study participant was 21.6%. This figure is higher than the prevalence reported in Addis Ababa (13%) and Mekele(14%) cities but lower than Ambo (27.5%) of Ethiopia[20, 21, 31]. The difference may be may be due to socio-demographic and economic differences between the study sites. The present study revealed that about 4.8% were overweighed. The this is greater than prevalence of overweight study conducted in Mekele (1.1%) and Ambo (2.6%) [20, 31] but lower than study conducted in Addis Ababa, 7.6% of adolescents were overweight [21]. About 1.1% of adolescents girls were obesity, which lower than study in Mekele city (0.2) and is slightly lower than study conducted in Addis Ababa (2.6%) and Ambo (1.7%) city [20, 21, 31].

Prevalence of stunting in these adolescent girls was 15.0%. This poor nutritional status of the girls remains uninterrupted throughout their adolescent life as stunting, is considered as index of chronic or long term duration of under nutrition, was observed during the entire period of adolescence. The study girls were considerably shorter than the reference population suggesting that they had not fully recuperated from childhood deficits. This figure is lower than study conducted in rural Tigray [19].

## Conclusion

The result of the study revealed that prevalence of anaemia, stunting and wasting/thinness, overweight and obesity is 32, 15, 21.6, 4.8 and 1.1% respectively. This indicates that appropriate intervention and focus should be given to these populations. As a nutritional status of adolescent girls contributes to the nutritional status of the community, there is a need to initiate intervention measures to improve the nutritional status of adolescent girls who are the future ‘mothers-to-be’. Hence, there is a need to create awareness among adolescents and their family about nutrition and health. Understanding the intergenerational effect of malnutrition in the study communities, there is a clear need for carefully designed longitudinal study to definitively answer the reasons for poor growth throughout the period of adolescence. Strategies addressing the nutritional status of girls are needed in addition to the conventional approach of providing services to pregnant and lactating women through the traditional maternal and child health care programs.
